# Effect of Estrogen on Musculoskeletal Performance and Injury Risk

**DOI:** 10.3389/fphys.2018.01834

**Published:** 2019-01-15

**Authors:** Nkechinyere Chidi-Ogbolu, Keith Baar

**Affiliations:** ^1^Biomedical Engineering Graduate Group, University of California, Davis, Davis, CA, United States; ^2^Department of Neurobiology, Physiology and Behavior, University of California, Davis, Davis, CA, United States; ^3^Department of Physiology and Membrane Biology, University of California, Davis, Davis, CA, United States

**Keywords:** estrogen, ACL, injury risk, exercise, tendon, ligament, muscle

## Abstract

Estrogen has a dramatic effect on musculoskeletal function. Beyond the known relationship between estrogen and bone, it directly affects the structure and function of other musculoskeletal tissues such as muscle, tendon, and ligament. In these other musculoskeletal tissues, estrogen improves muscle mass and strength, and increases the collagen content of connective tissues. However, unlike bone and muscle where estrogen improves function, in tendons and ligaments estrogen decreases stiffness, and this directly affects performance and injury rates. High estrogen levels can decrease power and performance and make women more prone for catastrophic ligament injury. The goal of the current work is to review the research that forms the basis of our understanding how estrogen affects muscle, tendon, and ligament and how hormonal manipulation can be used to optimize performance and promote female participation in an active lifestyle at any age.

## Introduction

Beyond its role as a sex hormone, estrogen has important roles in the development, maturation, and aging of extragonadal tissues such as bone (Hansen et al., 2009a; Cui et al., 2013; Ling-Ling et al., 2016), muscle (Dieli-Conwright et al., 2009; Enns and Tiidus, 2010), and connective tissues (Hansen et al., 2009a,b; Hansen and Kjaer, 2014; Hansen, 2018).

In young women, estrogen is produced from cholesterol in a series of reactions within the ovaries. The final reaction in the process is the conversion of testosterone to estradiol by the enzyme aromatase. In men and postmenopausal women, this reaction commonly occurs in adipose tissue which is high in aromatase activity (Nelson and Bulun, 2001). The most prevalent estrogen is 17β-estradiol with smaller amounts of estrone and estriol circulating as well (Heldring et al., 2007). As a steroidal hormone, estrogen can freely pass through the plasma membrane and move into the nucleus where it can bind to its nuclear receptors, the estrogen receptors (ER)α and β, and modify gene expression (Heldring et al., 2007). Beyond the nucleus, estrogen has a variety of post-transcriptional effects such as regulating the redox state of the cell (Kumar et al., 2010), altering mitochondrial function (Yao and Brinton, 2012), and directly inhibiting the activity of specific enzymes (Lee C. A. et al., 2015).

**Figure 1:**
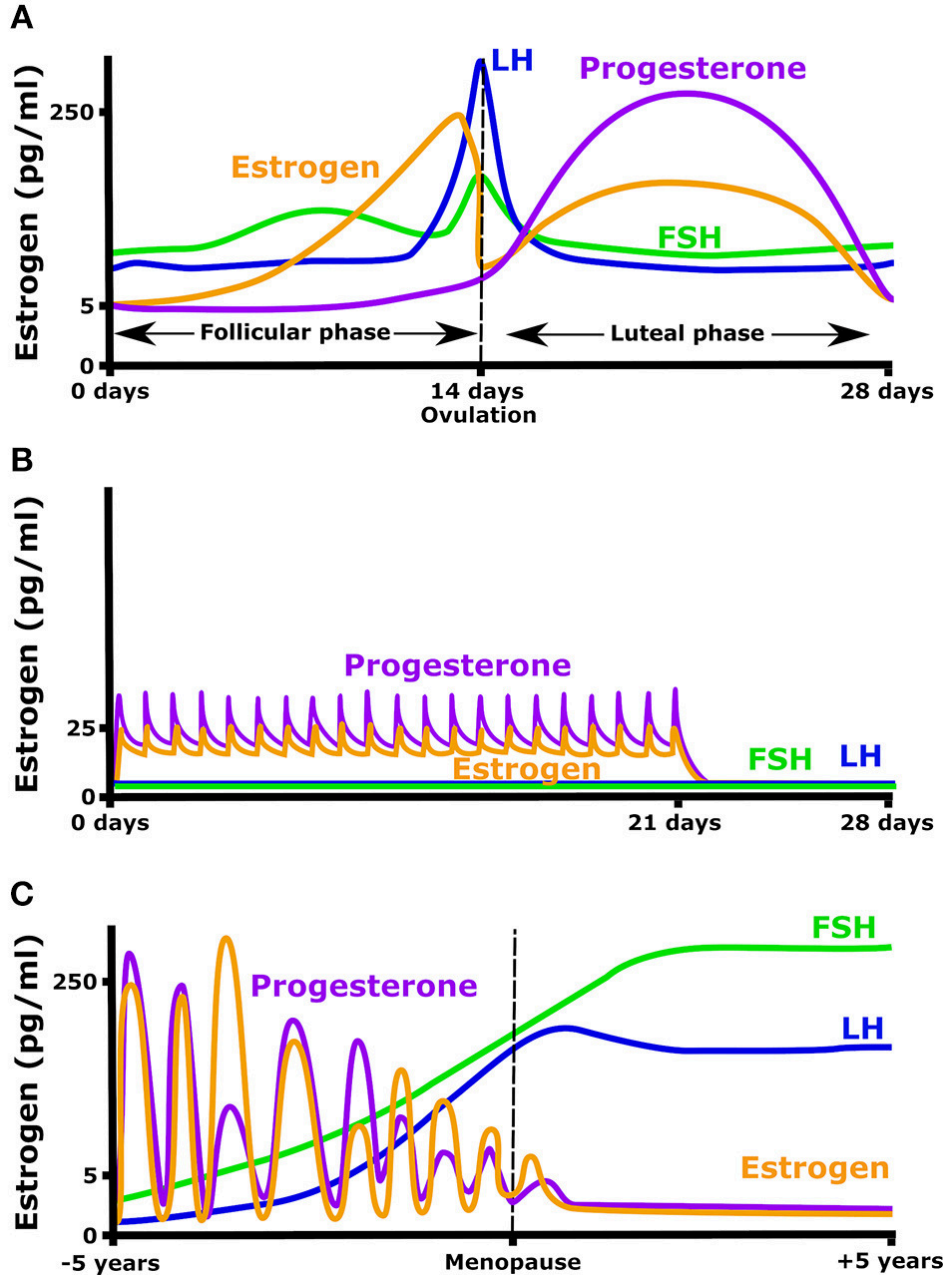
Hormonal fluctuation during **(A)** a normal menstrual cycle, **(B)** while taking an oral contraceptive (OC) containing both estrogen and progesterone, and **(C)** in the years before and after menopause.

Estrogen secretion naturally varies in young women, increasing 10- to 100-fold over the menstrual cycle. Beyond estrogen, the menstrual cycle is characterized by significant changes in other important plasma hormones such as follicle stimulating hormone (FSH), luteinizing hormone (LH), and progesterone (Figure 1). 17β-estradiol levels rise from 5 pg/ml at the early follicular phase, to a peak of 200–500 pg/ml just before ovulation. Ovulation is followed by a rapid decrease in estradiol, then estradiol, and progesterone both increase in the luteal phase giving a broad secondary peak. In order to prevent pregnancy, or simply to regulate hormone levels, many women take oral contraceptives that provide a daily low level of estrogen and progesterone. These pills typically maintain estradiol levels at ~25 pg/ml and decrease the ovulatory rise in estrogen (Mishell et al., 1972). This daily dose of estrogen and or progesterone also eliminates the cyclic rise in LH and FSH (Figure 1B). In the absence of oral contraceptives, the menstrual cycle will occur from puberty until menopause when menses stop, FSH and LH rise, and plasma estradiol and progesterone concentrations remain constantly low (Figure 1C).

Estrogen receptors are present in all musculoskeletal tissues including muscle (Barros and Gustafsson, 2011; Luo and Kim, 2016), bone (Cui et al., 2013), ligament (Liu et al., 1996), and tendon (Bridgeman et al., 2010). Within these tissues, estrogen is known to regulate metabolism (Nelson and Bulun, 2001), however, it is still unclear whether these effects are beneficial or harmful. Consistent with a role for estrogen in regulating musculoskeletal function, menstruating women suffer more ACL ruptures than men (Shultz et al., 2005, 2011), and menopause is characterized by increased risk of musculoskeletal injury (Enns and Tiidus, 2010), accelerated bone and muscle wasting (Rice et al., 1989; Frontera et al., 1991; Bassey et al., 1992; Häkkinen and Pakarinen, 1993), and decreased sensitivity to anabolic stimuli (Bamman et al., 2003; Teixeira et al., 2003). To counteract many of the negative aspects of menopause, hormone replacement therapy (HRT) has been used to reduce muscle and bone loss, and restore muscle protein balance (Hansen et al., 2012; Smith et al., 2014).

Given the sex differences in musculoskeletal injury risk and the growing number of active young women, the role of estrogen in musculoskeletal function is a burgeoning area of research. This review will highlight important developments, controversies, and unknowns in the relationship between estrogen and musculoskeletal function, with specific focus on muscle, tendon, and ligaments. There is a vast literature on the effects of estrogen on bone structure and function and therefore this topic will not be addressed in the current work. For an excellent review on this topic, see the recent review by Cauley et al. on estrogen and bone health in men and women (Cauley, 2015). This review will instead focus on the direct and indirect effects of estrogen on musculoskeletal function, as well as how these changes affect performance, adaptation, and injury risk in an active population.

## Estrogen and Muscle

Estrogen has a number of metabolic effects on skeletal muscle. When female animals lose estrogen through ovariectomy, mitochondrial function, membrane microviscosity, and complex I and I + III activities (Torres et al., 2018) all decrease. The loss of estrogen also results in increased mitochondrial H2O2 production (Valencia et al., 2016), decreased levels of antioxidant proteins such as glutathione peroxidase, catalase, and superoxide dismutase (Baltgalvis et al., 2010; Valencia et al., 2016), and impaired insulin sensitivity (Torres et al., 2018). These effects are due to the loss of estrogen since restoring normal estrogen levels restores cellular redox, and glucose homeostasis in skeletal muscle (Spangenburg et al., 2012; Camporez et al., 2013; Torres et al., 2018).

Beyond the metabolic roles, estrogen is clearly beneficial for muscle mass and strength, at least in animal models (McClung et al., 2006; Kitajima and Ono, 2016). For example, 24 weeks of estrogen deficiency resulted in a 10% decrease in strength that corresponded with an 18% decrease in CSA (Kitajima and Ono, 2016). Beyond the decrease in fiber CSA, ovariectomized (OVX) rats do not recover as well following unloading (McClung et al., 2006). In this example, following 14 days of unloading, OVX rats showed limited regrowth, and an increase in injured fibers during either 7 or 14 days of reloading. Supplementing the OVX rats with estradiol was enough to return fiber CSA and injured fiber numbers to control levels. These data suggest that in the absence of estrogen, muscle is more prone to injury, and this limits regrowth (McClung et al., 2006). Based on these and other data, Enns and Tiidus (2010) proposed that estrogen could stabilize the extracellular matrix or act as an antioxidant to decrease muscle injury; however, the effect of estrogen on human muscle has not been as clearly defined because shifts in estrogen are transient or associated with confounding differences in age, fitness level, and the type and intensity of exercise (Enns and Tiidus, 2010). Lastly, many studies looking to understand the role of estrogen on muscle function actually focus on sex differences, which goes far beyond simple changes in hormone levels.

In humans, much of the estrogen work has been performed in association with aging. Aging is a natural process that affects all aspects of life regardless of species. The goal of healthy aging is to slow the deterioration in physical and mental function as much as possible. Muscle protein turnover changes with age and this is further affected by sex. In postmenopausal women, higher rates of muscle protein synthesis and breakdown have been observed when compared to age matched men and premenopausal women (Smith et al., 2014). Even though higher rates of protein turnover might be expected to improve muscle quality, these women still experience a rapid decrease in muscle mass and strength, and as a result are more vulnerable to age-related frailty (Hansen and Kjaer, 2014). Muscle mass is largely dependent on the balance between the synthesis and degradation of muscle protein. The rapid decline in muscle mass after menopause therefore means either the increase in protein synthesis rate is counteracted by a greater increase in protein breakdown or that the proteins being synthesized are not the myofibrillar proteins but rather those needed for injury repair. Importantly, there is no significant sex difference observed in response to training and nutrition in middle-aged adults; however, postmenopausal women show reduced sensitivity to anabolic stimuli when compared to age-matched men (Bamman et al., 2003). This suggests that a chronic decrease in estrogen attenuates the response to anabolic stimuli (Hansen and Kjaer, 2014). In support of this hypothesis, when estrogen levels were raised to that of premenopausal women using estrogen replacement therapy (ERT), the response to anabolic stimuli was normalized (Hansen et al., 2012). Myofibrillar protein synthesis in women taking ERT is also increased in response to resistance exercise (Dieli-Conwright et al., 2009; Pöllänen et al., 2010), a response that is absent in postmenopausal women who do not take ERT (Pöllänen et al., 2010). These studies highlight the importance of estrogen in determining the sensitivity of muscle to anabolic signaling; however, more research is needed to understand whether monthly variations in estrogen have the same effect on anabolic signaling seen with chronic loss of estrogen.

One study that attempted to address this question in premenopausal women measured myofibrillar protein synthesis at the follicular (low estrogen and progesterone) and luteal (moderate estrogen and high progesterone) phases of the cycle (Miller et al., 2005). The authors found no significant difference in myofibrillar protein synthesis between phases (Miller et al., 2005). However, it should be noted that different subjects were tested in the follicular and luteal phases and subjects tested in the luteal phase came into the lab 4 days after ovulation (Miller et al., 2005). The result was that plasma estrogen was highly variable and the mean between the groups was only marginally (2-fold) higher, whereas progesterone levels were increased 40-fold, therefore, the luteal phase was more a measure of high progesterone than high estrogen (Miller et al., 2005). By contrast, oral contraceptives (OCs) provide a moderate, but relatively constant, level of estrogen with or without progesterone. Considering the fact that OC use alters regular hormone level fluctuations, this might be a good tool to understand how estrogen affects myofibrillar protein synthesis in response to anabolic stimuli. Exploiting this hormonal milieu, Hansen et al. (2011) found lower levels of myofibrillar protein FSR in users of one form of OC (35 μg ethinyl estradiol and 0.25 mg norgestimate/day), whereas a different formulation (30 μg ethinyl estradiol, and gestoden 0.0075 mg/day) had no effect of myofibrillar protein synthesis. Looking at the two different formulations would suggest that the 3,300% higher progesterone level may be more important in inhibiting muscle protein synthesis than the 16% difference in estrogen. Therefore, in young women the role of estrogen on muscle anabolism is still uncertain; however, it is clear that OCs with high progesterone have a negative impact on muscle.

Hormone replacement therapy (HRT) has been recommended as therapeutic for postmenopausal women to counteract some of the negative aspects of menopause (Enns and Tiidus, 2010). As far as the regulation of muscle performance, there are some clear benefits of HRT. In the most interesting study, habitual and maximal walking speed, thigh muscle composition, lower body muscle power (vertical jumping height), maximal isometric hand grip, and knee extension strengths were measured in 16 monozygotic twin pairs who were discordant for HRT use (one twin was on HRT while the other was not). Maximal walking speed and vertical jump height, thigh muscle CSA, and relative muscle area were larger in the HRT twins than their sisters. Habitual walking speed and maximal isometric strength were not significantly different between users and non-users. Importantly, serum estrogen levels were 5-fold higher in the twins on HRT, regardless of whether the product the women were taking contained only estrogen or estrogen and progesterone together (Ronkainen et al., 2009). In an earlier study, Sipilä et al. (2001), randomly assigned 80 postmenopausal women to 4 groups: exercise (Ex), HRT, exercise+HRT (ExHRT) or a no treatment control for a year. At the end of the intervention, the ExHRT group showed increases in muscle cross-sectional area (CSA; 7.1%), knee extension torque (8.3%), and vertical jump height (17.2%). A similar but smaller increase in vertical jump height (6.8%) and muscle CSA (6.3%) was observed in the HRT group. There was also a higher percentage of fat within the quadriceps muscle in the control group compared to the HRT and ExHRT groups (Sipilä et al., 2001). The lower fat mass could be a result of the correction of the lower LH/FSH ratio in postmenopausal women on HRT (Beydoun et al., 2012), or could be a metabolic consequence of the increase in muscle mass. Interestingly, exercise alone was less effective than HRT at maintaining muscle mass and function in these women. Together, these data suggest that HRT is beneficial for postmenopausal muscle mass and function, but that HRT together with exercise improves muscle mass and function more than either HRT or exercise alone.

A number of other studies have also addressed the role of estrogen replacement therapy on muscle mass and function (Taaffe et al., 2005; Hansen et al., 2012; Pingel et al., 2012; Smith et al., 2014). In a cross-sectional analysis of 840 postmenopausal women (259 on ERT and 581 controls), Taaffe et al. (2005) found that muscle cross-sectional area (CSA) and grip strength were greater in ERT users than in non-users (Taaffe et al., 2005). Hansen et al. (2012) surprisingly found that myofibrillar protein synthesis decreased with ERT suggesting that ERT inhibits basal skeletal muscle protein synthesis in the postabsorptive state. One interesting caveat was that following resistance exercise muscle protein synthesis increased significantly only in the ERT group (Hansen et al., 2012). These data suggest that ERT may decrease basal muscle protein synthesis while improving sensitivity to anabolic stimuli. In support of this hypothesis, Smith et al. (2014) found that fasting mixed muscle protein synthesis increased when postmenopausal women were given testosterone or progesterone, but not when given an acute estrogen injection. All together, the existing data suggest that acute treatment with estrogen does not improve basal muscle protein synthesis; however, estrogen increases the anabolic response to exercise and this may result in the increase in muscle mass reported in long term studies.

## Sinew: Tendon and Ligament

Within the musculoskeletal system, tendons, and ligaments (we will refer to these tissues collectively as sinew) function as connective tissues between bone and muscle and between bone and bone, respectively. In both tissues, the majority of the dry weight is collagen: 60–85% for tendon (Kjaer, 2004) and ~75% for ligament (Frank, 2004). Of this collagen, the majority is type I: 60% in tendon and up to 85% in a ligament. The mechanical properties of both the tendon and ligament are dependent on collagen fiber density, diameter, orientation, and cross-linking. The fibers can be cross-linked in two ways: enzymatically and non-enzymatically. Enzymatic cross-links are mediated largely by lysyl oxidase (LOX; Siegel, 1976; Siegel and Fu, 1976). By contrast, cross-links can be formed without a specific enzyme through a Maillard reaction between a sugar and an amino acid. These cross-links are called advanced glycation end-products (AGE), and as would be expected are higher in diabetics (Dyer et al., 1993). Both enzymatic cross-linking, through LOX, and non-enzymatic cross-linking through AGEs increase the stiffness of the tissues (Reddy et al., 2002; Svensson et al., 2013; Marturano et al., 2014). One of the main differences between enzymatic and non-enzymatic cross-links are their location and turnover rate, where AGEs decrease collagen turnover and over time this impairs sinew function (Hammes et al., 1991; Corman et al., 1998).

Since a ligament, such as the ACL within the knee, shows a direct relationship between laxity and rupture (Myer et al., 2008), a stiffer ligament is preferred to maintain joint stability and prevent injuries. Due to its role in connecting a compliant muscle to a stiff bone, a stiffer tendon is not always beneficial. In terms of performance, a stiff tendon transmits the force produced by a muscle to the bone faster and this can improve performance. However, when a tendon becomes too stiff this produces a strain concentration in muscle. What this means is that more of the strain (stretch) produced in a given movement is concentrated in the muscle that is connected to a stiff tendon than a muscle attached to a compliant tendon. In other words, instead of the tendon stretching while the muscle contracts isometrically (Griffiths, 1991), a stiff tendon doesn't stretch, and the muscle is forced to lengthen while contracting. The result is that a muscle attached to a stiff tendon will experience more eccentric load for a given movement. Since eccentric movements produce more muscle injury than concentric or isometric movements (Clarkson and Monica, 2002; LaStayo et al., 2003; Brockett et al., 2004), this means that muscles attached to stiff tendons will suffer more injury for a given movement than those attached to compliant tendons. Therefore, stiff ligaments are always better, stiff tendons can improve performance, but if the tendon becomes too stiff the associated muscle will suffer more injuries.

Interestingly, women suffer fewer muscle injuries, and more ligament ruptures than men (Arendt and Dick, 1995; Sewright et al., 2008; Hägglund et al., 2009; Edouard et al., 2016; Leblanc et al., 2017). These observations are consistent with lower sinew stiffness in women than men. Since knee laxity changes with estrogen levels through the menstrual cycle (Shultz et al., 2005), estrogen is believed to decrease sinew stiffness. Therefore, in the sections below, we will address how estrogen affects sinew mechanics and adaptation to loading.

## Estrogen and Ligament

**Figure 2:**
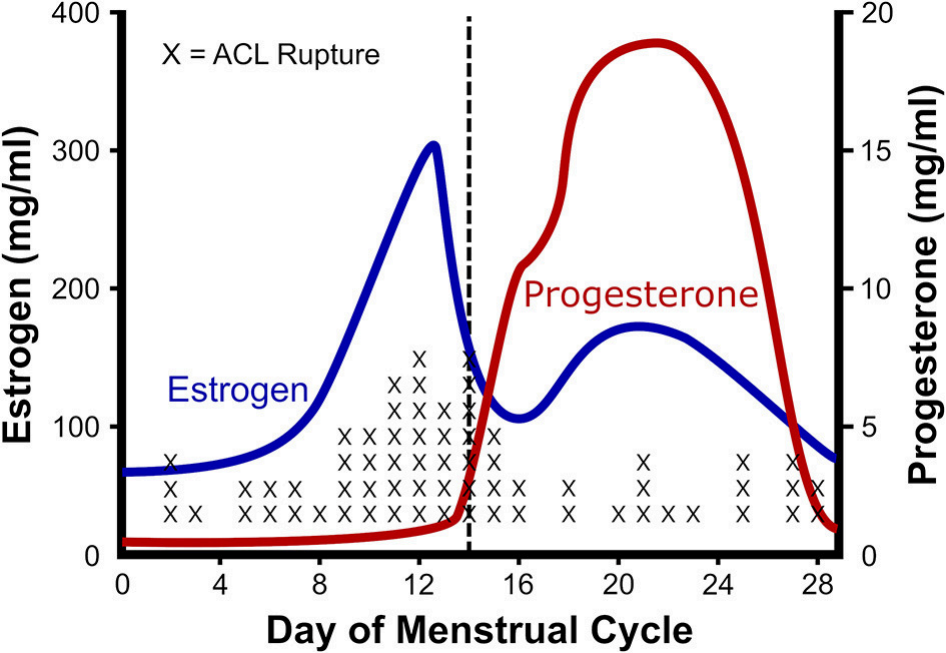
Relationship between estrogen and ACL rupture in a normal cycle. The rate of anterior cruciate ligament (ACL) rupture in relation to female hormones throughout a standard menstrual cycle. Note that with the ovulatory rise in estrogen there is a concomitant rise in ACL ruptures. Adapted from Wojtys et al. (2002).

One of the best characterized musculoskeletal differences between men and women, is the rupture rate of the anterior cruciate ligament (ACL). ACL ruptures occur 2–8 times more often among female athletes than their male counterparts (Arendt and Dick, 1995; Adachi et al., 2008). Given that there is a correlation between ACL injuries and knee laxity (Ramesh et al., 2005; Myer et al., 2008), and an association between knee laxity and the menstrual cycle (Deie et al., 2002; Shultz et al., 2005, 2010, 2011, 2012a,b), a number of groups have investigated the relationship between ACL injuries and phase of the menstrual cycle (Wojtys et al., 1998; Heitz et al., 1999; Carcia et al., 2004; Adachi et al., 2008; Lee et al., 2013; Lefevre et al., 2013; Herzberg et al., 2017). The resulting studies in general find a higher risk of ACL injury during the pre-ovulatory and ovulatory phases than luteal or follicular phases of the menstrual cycle (Beynnon et al., 2006; Ruedl et al., 2009; Lefevre et al., 2013). For example, Wojtys et al. (1998) and Wojtys et al. (2002) found higher risk (Wojtys et al., 1998) and occurrence of ACL injury in the ovulatory phase (Wojtys et al., 2002; Figure 2). To attempt to explain the increased ACL rupture in the pre-ovulatory phases, researchers have measured knee laxity throughout the cycle. In men and women with no history of knee injury, the men showed no statistical difference in knee laxity over time; however, in women laxity increased from 4.7 ± 0.8 mm in the follicular phase, to 5.3 ± 0.7 mm in the ovulatory phase (Deie et al., 2002). These authors concluded that knee laxity is dependent on female hormones (Deie et al., 2002). Similarly, Shultz et al. (2005) found that knee laxity increased in direct relation to elevations in plasma estradiol levels. The variations in laxity were found to be cyclic in nature. When estrogen concentration increased during the menstrual cycle, knee laxity increased as well (Shultz et al., 2010, 2011, 2012a). In fact, these authors found that knee laxity increased between 1 and 5 mm between the first day of menstruation and the day following ovulation, depending on estrogen levels. Lastly, Park et al. found a 17% decrease in knee stiffness during the ovulatory phase resulting in a change in knee laxity from 13.35 ± 2.53 mm during the follicular phase to 14.43 ± 2.60 mm during ovulation (Park et al., 2009). By contrast, Carcia et al. (2004) found no change in knee displacement in relation to cycle; however, it is important to note that that these authors used self-reported cycle length to estimate menstrual phase, whereas the other studies directly measured estrogen levels in concert with knee laxity. Since Myer et al. (2008) showed that for every 1.3 mm increase in knee displacement, risk of ACL injury goes up 4-fold, the rise in knee laxity reported by Deie, Park, and Shultz could explain the 2- to 8-fold higher rate of ACL rupture in women (Arendt and Dick, 1995; Adachi et al., 2008).

Since knee laxity changes with cycle phase, many active women want to know whether OCs could prevent the change in laxity and injury risk. In support of this idea, Gray et al. found that young women (aged 15–19) who undergo surgical repair of the ACL are 18% less likely to use oral contraceptives than matched controls (Gray et al., 2016). Further, Rahr-Wagner et al. found a 20% higher relative risk (RR) value of ACL injury in women who had never used OCs than in women who were long-term users (Rahr-Wagner et al., 2014). Together, these data suggest that ACL laxity changes through the cycle and eliminating the changes in estrogen using oral contraceptives decreases the risk of ACL rupture.

**Figure 3:**
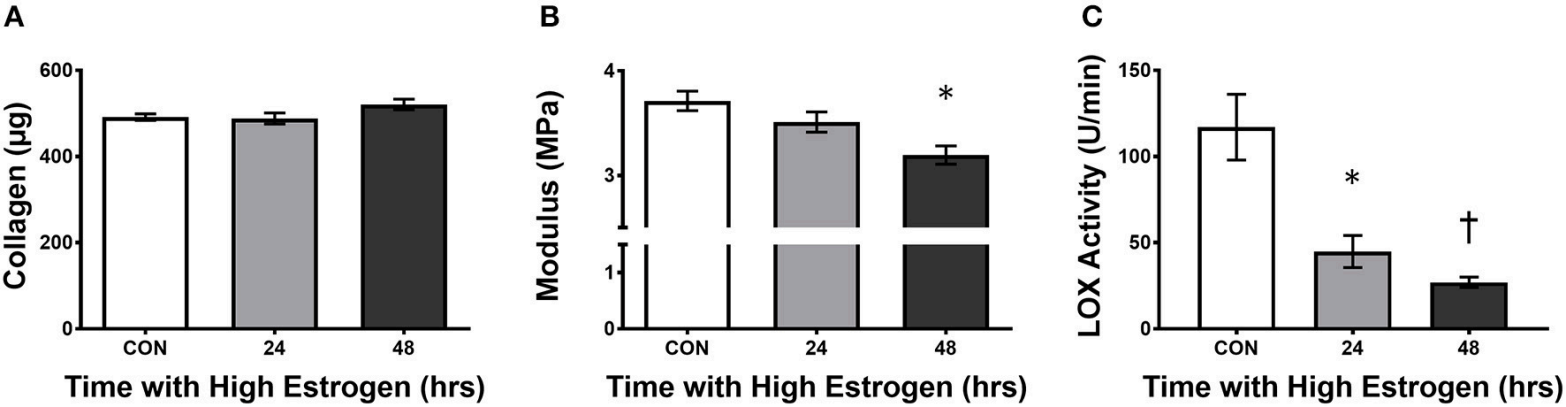
High estrogen decreasing engineered ligament stiffness due to inhibition of lysyl oxidase**. (A)** Collagen content, **(B)** tangent modulus, and **(C)** lysyl oxidase (LOX) activity in ligaments engineered from human ACL cells isolated from women following 24 or 48 h of treatment of the constructs with physiologically high (500 pg/ml) of estrogen. Note that even though there is a slight rise in collagen, the stiffness of the grafts decreases concomitant with an increase in estrogen in the media. *indicates different than control (*p* < 0.05), whereas ^†^indicates different than 24 h (*p* < 0.05). Adapted from Lee C. A. et al. (2015).

With evidence pointing to hormonal fluctuations of the menstrual cycle having an influence on ligament injury risk, the question of how sex hormones, estrogen in particular, increase the risk of injury has been the focus of study. In cell culture, Yu et al. (1999) found an early increase in proliferation and procollagen synthesis in freshly isolated ACL cells that were incubated with estrogen (Lee H. et al., 2015). However, the benefit of estrogen becomes less apparent with time in culture (Lee H. et al., 2015). In support, Chen et al. (2014) found an estrogen dose-dependent increase in proliferation of cells from the *ligamentum flavum* that lasted only 24 h in culture (Chen et al., 2014). Although expression of collagen mRNA didn't change significantly, there was a decrease in the ratio of collagen to elastin at the protein level after the cells were treated with 17β-estradiol. The authors attributed this shift in protein to the up regulation of matrix metalloproteinase 13 (MMP-13) which degrades collagen but not elastin (Chen et al., 2014). This suggests that estrogen could decrease collagen protein and in the case of lumbar stenosis, prevent hypertrophy of the ligamentum flavum, and reduce risk of the disease (Chen et al., 2014). However, the effect of estrogen on collagen synthesis in ligaments has yielded conflicting results in other systems. Some studies suggest that estradiol has a negative effect on collagen synthesis (Hama et al., 1976; Liu et al., 1997), whereas others saw positive effects (Lee et al., 2004a,b; Lee C. A. et al., 2015) and still others saw no effect (Seneviratne et al., 2004; Mamalis et al., 2011). Hama et al. (1976) found decreased collagen content in the capsular ligament with estrogen administration to ovariectomized rats (Hama et al., 1976), however Lee et al. (2004a,b) found increased collagen synthesis (Lee et al., 2004b) with a corresponding increase in Type I collagen mRNA (Lee et al., 2004a). Liu et al. (1997) also found decreased Type I collagen synthesis at physiological estradiol levels in monolayer culture of fibroblasts derived from rabbit ACL (Liu et al., 1997); however, in 3D ligaments engineered from human ACL cells, high estrogen results in increased collagen accumulation within the grafts (Lee C. A. et al., 2015). Despite conflicting results on fibroblast proliferation and collagen synthesis, there is a general consensus that the mechanical strength of the tissue decreases. In the engineered ligaments, despite increased collagen content, the mechanical properties of the tissue (UTS and modulus) decreased due to the inhibition of lysyl oxidase activity by estrogen (Lee C. A. et al., 2015). In these experiments, treating engineered ligaments with physiologically high estrogen for 48 h resulted in an 80% decrease in lysyl oxidase activity without changing LOX expression (Figure 3). These data suggest that estrogen may increase collagen synthesis or incorporation but decrease sinew stiffness by directly inhibiting lysyl oxidase and decreasing cross-linking.

## Estrogen and Tendon

If estrogen decreases lysyl oxidase activity in sinews, this would be expected to decrease tendon stiffness, and therefore decrease the incidence of injury to the associated muscles. In fact, as mentioned above women suffer fewer muscle injuries than men (Hägglund et al., 2009; Edouard et al., 2016). In professional soccer, women suffer 54% fewer muscle strains than their male counterparts (Hägglund et al., 2009). The majority of the benefit results from decreases in groin (83% fewer) and hamstring (36% fewer) pulls. A decrease in tendon stiffness could also leave the tendon less prone to injury. In fact, women are at lower risk of sustaining an Achilles' tendon rupture than men until menopause, after which the risk becomes similar in both sexes (Hansen and Kjaer, 2014, 2016). Use of OCs (which maintain moderate estrogen levels) has been linked with increased risk of Achilles tendinopathy (Holmes and Johnny, 2006), indicating that the periodic rise in estrogen to physiologically high levels may be needed to decrease Achilles injury. Similarly, OCs have been linked with greater muscle damage and delayed onset muscle soreness after exercise (Savage and Priscilla, 2002; Lee H. et al., 2015; Minahan et al., 2015). As discussed above, an increase in muscle damage is consistent with an increase in tendon stiffness that decreases shielding of the muscle from strain injury. Therefore, periodic rises in estrogen levels are necessary for the protective effect on tendon and muscle health.

There have been a number of elegant studies performed in women that have tried to establish the mechanism underlying the effect of estrogen on tendon health. Many of these studies have focused on collagen synthesis and the interactions between estrogen and exercise. Interestingly, the studies have contrasting results depending on age—premenopausal women compared to postmenopausal women—even when they come from the same research group. In premenopausal women, holding estrogen levels constant with oral contraceptives resulted in decreased exercise stimulated collagen synthesis (Lee et al., 2004b; Miller et al., 2006; Magnusson et al., 2007; Hansen et al., 2008, 2009a; Westh et al., 2008; Kjaer et al., 2009). In the first of these studies, a group taking oral contraceptives containing moderate estradiol was compared to non-OC users in the follicular phase, when estrogen levels are naturally low, both at rest and following 1 h of kicking exercise. Patellar tendon collagen synthesis, measured 24 h after exercise using microdialysis to capture the N-terminal peptide of procollagen I (PINP), was not significantly different at rest; however, following exercise the women taking OC showed no change in collagen synthesis whereas the control women doubled PINP production (Hansen et al., 2008). The same group repeated the study using stable isotope labeled proline and patellar tendon biopsies to detect the incorporation of newly synthesized collagen into the tendon (Hansen et al., 2009a). In contrast to the microdialysis experiment, OC use decreased resting collagen synthesis, and neither group saw an increase in collagen incorporation into the patellar tendon after exercise (Hansen et al., 2009a). This is in contrast to men where the same 1 h kicking exercise increased new collagen incorporation 70% by 24 h (Miller et al., 2005). Consistent with the stable isotope data from Hansen et al. (2009a), when the same group compared the data in men to an equivalent cohort of women, tendon collagen synthesis was 46% lower in the women at rest and was unaffected by exercise (Miller et al., 2007). Together, these data suggest that in young active women, the incorporation of new collagen into the patellar tendon is lower and does not increase following exercise. This does not mean that woman are synthesizing less collagen. The PINP data suggests that women synthesize more collagen in response to exercise; however, this collagen may not be incorporated into the tendon to the same degree in women. In support of this hypothesis, Laurent (1987) showed that in muscle 49% of newly produced collagen is degraded rapidly before it is incorporated. This raises the possibility that estrogen differentially regulates the synthesis and incorporation of collagen into the matrix of the sinew.

**Figure 4:**
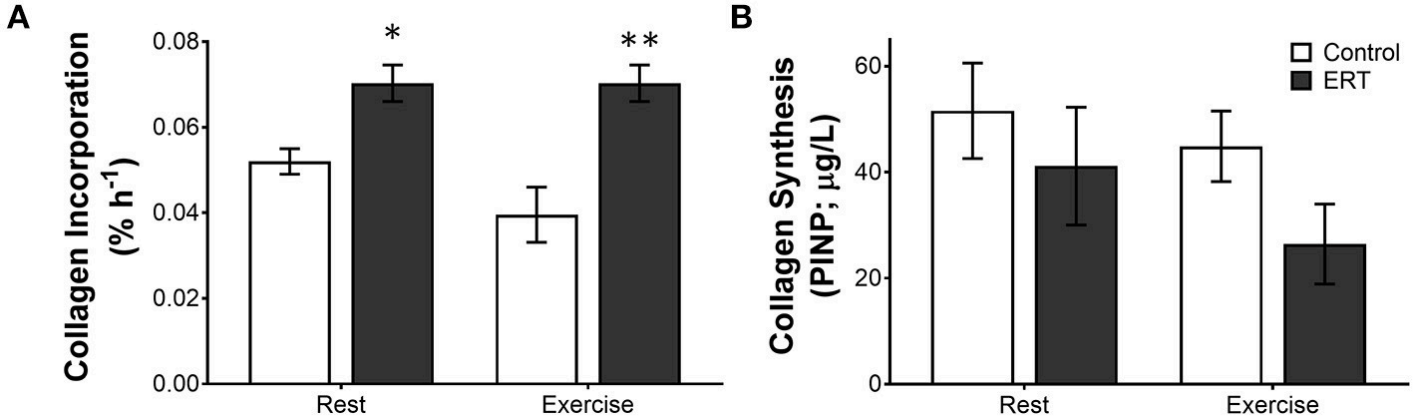
Differential measures of collagen incorporation and synthesis with estrogen replacement and exercise. The rate of **(A)** collagen incorporation of proline into the patellar tendon or **(B)** the appearance of the N-terminal propeptide of collagen I in post-menopausal women ± estrogen replacement therapy (ERT) and exercise. Note that with ERT collagen incorporation is higher in the same women where collagen synthesis is repressed. Further, exercise tends to decrease collagen incorporation and synthesis in controls, whereas ERT users show no effect on incorporation or a large drop in collagen synthesis. Symbols (*P < 0.01, **P < 0.001) show significance determined by unpaired *t*-test Control vs. ERT. These data suggest that there is a large methodological discrepancy between the two measures. Adapted from Hansen et al. (2009b).

In premenopausal women, a consistent moderate level of estrogen from OC decreases collagen synthesis; however, in postmenopausal women, estrogen replacement therapy, which provides a daily moderate rise in estrogen, is linked with increased tendon collagen synthesis (Hansen et al., 2009b). In postmenopausal women, collagen incorporation into the patellar tendon was 47% higher in ERT users compared with control (Hansen et al., 2009b). Even though ERT boosted collagen incorporation at rest, exercise did not increase collagen incorporation further (Finni et al., 2009; Hansen et al., 2009b). Interestingly though, using the PINP measure at the same time, Hansen et al. saw more collagen synthesis in the tendons of the control women than the ERT users (Figure 4), again suggesting that estrogen effects collagen incorporation into tendons differently than collagen synthesis itself (Hansen et al., 2009b). As to which measure is the best indicator of long term tendon structure/function, in the monozygotic twin study discussed above, the twins on HRT had a smaller Achilles tendon CSA when compared to the twins who did not take HRT (Finni et al., 2009) and a similar decrease in Achilles CSA was found in a separate study in a larger group of active women taking HRT (Cook et al., 2007). Together, these data suggest that the decrease in PINP in the microdiasylate of a tendon may better represent the long term changes in tendon structure/function than the increased incorporation of stable isotopes. One reason that the PINP measure may better reflect long term changes is that the current stable isotope techniques are highly dependent on delivery of the isotope to the tissue in a limited amount of time. Since tendon is relatively avascular, this may not be the best way to measure tendon turnover. However, with the ability to measure stable water incorporation into tissues over a much longer time frame, new isotope techniques could vastly improve our understanding of the dynamics of these tissues.

In an effort to gain a better mechanistic understanding of how estrogen can increase collagen content while decreasing tendon mechanics in young women, researchers have turned to animal, and cell culture models. Creating an estrogen deficiency in rats using ovariectomy results in a 28% decrease in collagen content in Achilles tendon (Ramos et al., 2012). When OVX rats are treated with genistein, a natural isoflavone phytoestrogen, collagen content within the Achilles is returned to control levels (Ramos et al., 2012). Interestingly, unlike native estrogen that decreases tendon stiffness, genistein showed no effect on mechanical properties of the Achilles (Ramos et al., 2012), suggesting that phytoestrogens produce the increase in collagen without the negative effect on stiffness. In 2D cultured Achilles tendon cells, Irie et al. (2010) found that estrogen or a selective estrogen receptor modulator (SERM) increases the expression of MMP-13, suggesting that estrogen could increase the rate of collagen turnover. As mentioned above, in our tissue engineered model that allows us to determine both collagen content and mechanics, collagen content increased significantly with increasing estrogen in the media; however, as with *in vivo* sinew the tissue stiffness decreased (Lee C. A. et al., 2015). We have yet to determine whether the increase in collagen content was the result of a change in collagen synthesis or incorporation (Lee C. A. et al., 2015); however, the decrease in stiffness correlated with a decrease in LOX activity.

One interesting possible explanation for how estrogen could increase collagen content is related to an indirect effect on insulin-like growth factor (IGF)-1. Both in humans (Hansen et al., 2013) and in engineered ligaments (West et al., 2015) the administration of IGF-1 increases tendon collagen synthesis. In humans, both the incorporation of collagen into the patellar tendon and the local production of PINP were significantly increased with local IGF-1 administration (Hansen et al., 2013), suggesting that IGF-1 can enhance tendon collagen synthesis and incorporation. Estrogen directly modulates both IGF-1 and IGF binding proteins (Hansen et al., 2009b) and can therefore mediate its positive effects through an increase in IGF-1 signaling. IGF-1 in turn can affect collagen content through an increase in protein synthesis through the production of the La-related protein (LARP) 6 (Blackstock et al., 2014). LARP6 is a binding protein that is increased by IGF-1, directly binds to type I collagen mRNA, and specifically increases the translation of type I collagen.

## Practical Considerations to Maximize Performance and Minimize Injury

Given the sometimes confusing data on the role of estrogen in musculoskeletal function, the question many active women have is: “based on our current knowledge, what recommendations can be made for how to maximize musculoskeletal function?” From the data discussed above, it appears that like many other performance strategies, in young women hormonal cycling is something that needs to be handled differently depending on the phase of training. For young women who are not competing in anything at a high level, normal cycling is beneficial for musculoskeletal health, and performance. In this population, the benefits of high estrogen on the anabolic response to exercise in muscle and tendon and improved muscle repair means that over time these women will have stronger muscles, tendons, and bones if they allow for the periodic rise of estrogen that occurs before ovulation. In competitive athletes, the benefits of normal cycling can be seen by contrasting them with those athletes who experience relative energy deficiency in sport (RED-S), formerly known as the female athlete triad (Heikura et al., 2017). With a chronic energy deficiency, women stop normal cycling, and estrogen levels drop to very low levels, resulting in amenorrhea, loss of bone mass, and increased risk of musculoskeletal injury (Heikura et al., 2017). Again, because of the beneficial effects on muscle, tendon, and bone, competitive athletes should look to maintain their normal cycling when they are looking to maximize their adaptation to training: during the offseason or in the base phase of their training. As they begin to shift into the season, or during the specific preparation phase of training, they should consider taking an oral contraceptive that contains low levels of progesterone. The low level of estrogen in the OC would decrease the negative effects of the ovulatory rise in estrogen on tendon and ligament mechanics (Lee C. A. et al., 2015), whereas the work of Hansen et al. (2011) showed that only high progesterone OCs decrease muscle protein synthesis. In this way, training would be performed in the absence of OCs and therefore lower tendon stiffness, and induce higher anabolic responses to training and maximal muscle repair on hard days. This would result in fewer muscle pulls and a greater metabolic cost of training, increasing the stimulus for adaptation and the likelihood of a healthy build up phase. Shifting to the low progesterone OC in the specific preparation phase, or in season, would help increase stiffness within tendon and ligament while not preventing muscle repair following quality sessions or games. The result would be high rate of force development resulting in better performance and a lower risk of musculoskeletal injuries during the competitive season. However, it should be noted that this strategy would leave the athlete at a greater risk for catastrophic injury for ~5 days a month during training. Therefore, novel strategies to prevent the negative effects of estrogen on joint laxity are desperately needed to decrease the risk of catastrophic injuries in active women.

In postmenopausal women, the strategy is less clear. In this population, hormone replacement improves muscle mass and function by improving muscle repair, and the response to feeding and exercise. Bone mass and function is also improved by HRT (Zhao et al., 2015). The problem is that long term HRT use is associated with decreased tendon cross-sectional area, especially in an active population (Cook et al., 2007). The result may be a bigger, stronger muscle pulling on a small brittle tendon that is in turn connected to a stiffer bone. This would result in increased impedance mismatch, differences in stiffness between connected tissues, that can produce strain concentrations, and promote injury. However, not taking HRT would accelerate sarcopenia and osteoporosis. Therefore, to date the data suggest that HRT is beneficial for musculoskeletal function in postmenopausal women, but extra care should be taken to maximize tendon function. What is really lacking for these women is a way to get the positive effects of estrogen on muscle and bone repair and anabolic responses to loading and nutrition without the negative long term effects on tendon. Phytoestrogens may provide some hope, but much further work is needed to establish the efficacy of these natural products.

## Conclusions and Future Research

It is clear that estrogen has a dramatic effect on musculoskeletal function. In the past, much of the research focus has been on the strong connection between estrogen and bone. However, recently the effect of estrogen on other musculoskeletal tissues such as muscle, tendon, and ligament has become the focus of more research. These studies make it clear that estrogen improves muscle proteostasis and increases sinew collagen content; however, the benefits on bone, and muscle come at the cost of decreased connective tissue stiffness. Evolutionarily, this makes sense since laxer joints and better repair following injury would facilitate healthy childbirth and recovery. However, as more women participate in sports it is clear that these physiological effects of estrogen contribute to decreases in power and performance and make women more prone for catastrophic ligament injury. In order to promote female participation in an active lifestyle throughout their life span, more research is needed to determine how nutrition, training, and hormonal manipulation can be used to promote optimal performance at any age.

## Author Contributions

All authors listed have made a substantial, direct and intellectual contribution to the work, and approved it for publication.

### Conflict of Interest Statement

The authors declare that the research was conducted in the absence of any commercial or financial relationships that could be construed as a potential conflict of interest.

## References

[B1] Adachi N. Nawata K. Maeta M. Kurozawa Y. (2008). Relationship of the menstrual cycle phase to anterior cruciate ligament injuries in teenaged female athletes. Arch. Orthop. Trauma Surg. 128, 473–478. 10.1007/s00402-007-0461-1 17909824

[B2] Arendt E. Dick R. (1995). Knee injury patterns among men and women in collegiate basketball and soccer: NCAA data and review of literature. Am. J. Sports Med. 23, 694–701. 10.1177/036354659502300611 8600737

[B3] Baltgalvis K. A. Greising S. M. Warren G. L. Lowe D. A. (2010). Estrogen regulates estrogen receptors and antioxidant gene expression in mouse skeletal muscle. PLoS ONE 5:e10164. 10.1371/journal.pone.0010164 20405008 PMC2854140

[B4] Bamman M. M. Hill V. J. Adams G. R. Haddad F. Wetzstein C. J. Gower B. A.. (2003). Gender differences in resistance-training-induced myofiber hypertrophy among older adults. J. Gerontol. Ser. A Biol. Sci. Med. Sci. 58, B108–B116. 10.1093/gerona/58.2.B108 12586847

[B5] Barros R. P. Gustafsson J. Å. (2011). Estrogen receptors and the metabolic network. Cell Metabol. 14, 289–299. 10.1016/j.cmet.2011.08.005 21907136

[B6] Bassey E. J. Fiatarone M. A. O'Neill E. F. Kelly M. Evans W. J. Lipsitz L. A. (1992). Leg extensor power and functional performance in very old men and women. Clin. Sci. 82, 321–327. 10.1042/cs0820321 1312417

[B7] Beydoun H. A. Beydoun M. Wiggins N. Stadtmauer L. (2012). Relationship of obesity-related disturbances with LH/FSH ratio among post-menopausal women in the United States. Maturitas 71, 55–61. 10.1016/j.maturitas.2011.10.010 22088801 PMC3398813

[B8] Beynnon B. D. Johnson R. J. Braun S. Sargent M. Bernstein I. M. Skelly J. M.. (2006). The relationship between menstrual cycle phase and anterior cruciate ligament injury: a case-control study of recreational alpine skiers. Am. J. Sports Med. 34, 757–764. 10.1177/0363546505282624 16436538

[B9] Blackstock C. D. Higashi Y. Sukhanov S. Shai S. Y. Stefanovic B. Tabony A. M.. (2014). Insulin-like growth factor-1 increases synthesis of collagen type I via induction of the mRNA-binding protein LARP6 expression and binding to the 5′ stem-loop of COL1a1 and COL1a2 mRNA. J. Biol. Chem. 289, 7264–74. 10.1074/jbc.M113.518951 24469459 PMC3953245

[B10] Bridgeman J. T. Zhang Y. Donahue H. Wade A. M. Juliano P. J. (2010). Estrogen receptor expression in posterior tibial tendon dysfunction: a pilot study. Foot Ankle Int. 31, 1081–1084. 10.3113/FAI.2010.1081 21189209

[B11] Brockett C. L. David L. M. Proske U. W. E. (2004). Predicting hamstring strain injury in elite athletes. Med. Sci. Sports Exerc. 36, 379–387. 10.1249/01.MSS.0000117165.75832.05 15076778

[B12] Camporez J. P. Jornayvaz F. R. Lee H. Y. Kanda S. Guigni B. A. Kahn M.. (2013). Cellular mechanism by which estradiol protects female ovariectomized mice from high-fat diet-induced hepatic and muscle insulin resistance. Endocrinology 154, 1021–1028. 10.1210/en.2012-1989 23364948 PMC3578999

[B13] Carcia C. R. Shultz S. J. Granata K. P. Gansneder B. M. Perrin D. H. (2004). Knee ligament behavior following a controlled loading protocol does not differ by menstrual cycle day. Clin. Biomech. 19, 1048–1054. 10.1016/j.clinbiomech.2004.07.006 15531055

[B14] Cauley J. A. (2015). Estrogen and bone health in men and women. Steroids 99, 11–15. 10.1016/j.steroids.2014.12.010 25555470

[B15] Chen M. H. Hu C. K. Chen P. R. Chen Y. S. Sun J. S. Chen M. H. (2014). Dose-dependent regulation of cell proliferation and collagen degradation by estradiol on ligamentum flavum. BMC Musculoskel. Disord. 15:238. 10.1186/1471-2474-15-238 25022571 PMC4108226

[B16] Clarkson P. M. Monica J. H. (2002). Exercise-induced muscle damage in humans. Am. J. Phys. Med. Rehabil. 81, S52–S69. 10.1097/00002060-200211001-00007 12409811

[B17] Cook J. L. Bass S. L. Black J. E. (2007). Hormone therapy is associated with smaller Achilles tendon diameter in active post-menopausal women. Scand. J. Med. Sci. Sports 17, 128–132. 10.1111/j.1600-0838.2006.00543.x 17394473

[B18] Corman B. Duriez M. Poitevin P. Heudes D. Bruneval P. Tedgui A.. (1998). Aminoguanidine prevents age-related arterial stiffening and cardiac hypertrophy. Proc. Natl. Acad. Sci. 95, 1301–1306. 10.1073/pnas.95.3.1301 9448326 PMC18752

[B19] Cui J. Shen Y. Li R. (2013). Estrogen synthesis and signaling pathways during aging: from periphery to brain. Trends Mol. Med. 19, 197–209. 10.1016/j.molmed.2012.12.007 23348042 PMC3595330

[B20] Deie M. Sakamaki Y. Sumen Y. Urabe Y. Ikuta Y. (2002). Anterior knee laxity in young women varies with their menstrual cycle. Int. Orthop. 26, 154–156. 10.1007/s00264-001-0326-0 12073107 PMC3620888

[B21] Dieli-Conwright C. M. Spektor T. M. Rice J. C. Sattler F. R. Todd Schroeder E. (2009). Influence of hormone replacement therapy on eccentric exercise induced myogenic gene expression in postmenopausal women. J. Appl. Physiol. 107, 1381–1388. 10.1152/japplphysiol.00590.2009 19696363 PMC2777804

[B22] Dyer D. G. Dunn J. A. Thorpe S. R. Bailie K. E. Lyons T. J. McCance D. R.. (1993). Accumulation of Maillard reaction products in skin collagen in diabetes and aging. J. Clin. Invest. 91, 2463–2469. 10.1172/JCI116481 8514858 PMC443306

[B23] Edouard P. Branco P. Alonso J. M. (2016). Muscle injury is the principal injury type and hamstring muscle injury is the first injury diagnosis during top-level international athletics championships between 2007 and 2015. Br. J. Sports Med. 50, 619–630. 10.1136/bjsports-2015-095559 26887415

[B24] Enns D. L. Tiidus P. M. (2010). The influence of estrogen on skeletal muscle. Sports Med. 40, 41–58. 10.2165/11319760-000000000-00000 20020786

[B25] Finni T. Kovanen V. Ronkainen P. H. Pöllänen E. Bashford G. R. Kaprio J.. (2009). Combination of hormone replacement therapy and high physical activity is associated with differences in Achilles tendon size in monozygotic female twin pairs. J. Appl. Physiol. 106, 1332–1337. 10.1152/japplphysiol.91439.2008 19164771

[B26] Frank C. B. (2004). Ligament structure, physiology and function. J. Musculoskel. Neuronal Interact. 4, 199–201. 15615126

[B27] Frontera W. R. Hughes V. A. Lutz K. J. Evans W. J. (1991). A cross-sectional study of muscle strength and mass in 45-to 78-yr-old men and women. J. Appl. Physiol. 71, 644–650. 10.1152/jappl.1991.71.2.644 1938738

[B28] Gray A. M. Zbgniew G. Jacques G. B. (2016). Effects of oral contraceptive use on anterior cruciate ligament injury epidemiology. Med. Sci. Sports Exerc. 48, 648–654. 10.1249/MSS.0000000000000806 26540261

[B29] Griffiths R. I. (1991). Shortening of muscle fibres during stretch of the active cat medial gastrocnemius muscle: the role of tendon compliance. J. Physiol. 436, 219–236. 10.1113/jphysiol.1991.sp018547 2061831 PMC1181502

[B30] Hägglund M. Markus W. Ekstrand J. (2009). Injuries among male and female elite football players. Scand. J. Med. Sci. Sports 19, 819–827. 10.1111/j.1600-0838.2008.00861.x 18980604

[B31] Häkkinen K. Pakarinen A. (1993). Muscle strength and serum testosterone, cortisol and SHBG concentrations in middle-aged and elderly men and women. Acta Physiol. Scand. 148, 199–207. 10.1111/j.1748-1716.1993.tb09549.x 8352031

[B32] Hama H. Yamamuro T. Takeda T. (1976). Experimental studies on connective tissue of the capsular ligament: influences of aging and sex hormones. Acta Orthop. Scand. 47, 473–479. 10.3109/17453677608988723 998180

[B33] Hammes H. P. Martin S. Federlin K. Geisen K. Brownlee M. (1991). Aminoguanidine treatment inhibits the development of experimental diabetic retinopathy. Proc. Natl. Acad. Sci. 88, 11555–11558. 10.1073/pnas.88.24.11555 1763069 PMC53174

[B34] Hansen M. (2018). Female hormones: do they influence muscle and tendon protein metabolism?. Proc. Nutr. Soc. 77, 32–41. 10.1017/S0029665117001951 28847313

[B35] Hansen M. Boesen A. Holm L. Flyvbjerg A. Langberg H. Kjaer M. (2013). Local administration of insulin-like growth factor-I (IGF-I) stimulates tendon collagen synthesis in humans. Scand. J. Med. Sci. Sports 23, 614–619. 10.1111/j.1600-0838.2011.01431.x 22288768

[B36] Hansen M. Kjaer M. (2014). Influence of sex and estrogen on musculotendinous protein turnover at rest and after exercise. Exerc. Sport Sci. Rev. 42, 183–192. 10.1249/JES.0000000000000026 25062001

[B37] Hansen M. Kjaer M. (2016). Sex hormones and tendon, Metabolic Influences on Risk for Tendon Disorders, ed Ackermann P. Hart D. A. (Cham: Springer), 139–149. 10.1007/978-3-319-33943-6_13 27535256

[B38] Hansen M. Kongsgaard M. Holm L. Skovgaard D. Magnusson S. P. Qvortrup K.. (2009b). Effect of estrogen on tendon collagen synthesis, tendon structural characteristics, and biomechanical properties in postmenopausal women. J. Appl. Physiol. 106, 1385–1393. 10.1152/japplphysiol.90935.2008 18927264

[B39] Hansen M. Koskinen S. O. Petersen S. G. Doessing S. Frystyk J. Flyvbjerg A.. (2008). Ethinyl oestradiol administration in women suppresses synthesis of collagen in tendon in response to exercise. J. Physiol. 586, 3005–3016. 10.1113/jphysiol.2007.147348 18420709 PMC2517199

[B40] Hansen M. Langberg H. Holm L. Miller B. F. Petersen S. G. Doessing S.. (2011). Effect of administration of oral contraceptives on the synthesis and breakdown of myofibrillar proteins in young women. Scand. J. Med. Sci. Sports 21, 62–72. 10.1111/j.1600-0838.2009.01002.x 19883384

[B41] Hansen M. Miller B. F. Holm L. Doessing S. Petersen S. G. Skovgaard D.. (2009a). Effect of administration of oral contraceptives *in vivo* on collagen synthesis in tendon and muscle connective tissue in young women. J. Appl. Physiol. 106, 1435–1443. 10.1152/japplphysiol.90933.2008 18845777

[B42] Hansen M. Skovgaard D. Reitelseder S. Holm L. Langbjerg H. Kjaer M. (2012). Effects of estrogen replacement and lower androgen status on skeletal muscle collagen and myofibrillar protein synthesis in postmenopausal women. J. Gerontol. Ser. A Biomed. Sci. Med. Sci. 67, 1005–1013. 10.1093/gerona/gls007 22389460

[B43] Heikura I. A. Uusitalo A. L. T. Stellingwerff T. Bergland D. Mero A. A. Burke L. M. (2017). Low energy availability is difficult to assess but outcomes have large impact on bone injury rates in elite distance athletes. Int. J. Sport Nutr. Exerc. Metab. 28, 403–411. 10.1123/ijsnem.2017-0313 29252050

[B44] Heitz N. A. Eisenman P. A. Beck C. L. Walker J. A. (1999). Hormonal changes throughout the menstrual cycle and increased anterior cruciate ligament laxity in females. J. Athl. Train. 34:144–149. 16558557 PMC1322903

[B45] Heldring N. Pike A. Andersson S. Matthews J. Cheng G. Hartman J.. (2007). Estrogen receptors: how do they signal and what are their targets. Physiol. Rev. 87, 905–931. 10.1152/physrev.00026.2006 17615392

[B46] Herzberg S. D. Motu'apuaka ML Lambert, W. Fu R. Brady J. Guise J-M. (2017). The effect of menstrual cycle and contraceptives on ACL injuries and laxity: a systematic review and meta-analysis. Orthop. J. Sports Med. 5:2325967117718781. 10.1177/2325967117718781 28795075 PMC5524267

[B47] Holmes G. B. Johnny L. (2006). Etiologic factors associated with symptomatic achilles tendinopathy. Foot Ankle Int. 27, 952–959. 10.1177/107110070602701115 17144959

[B48] Irie T. Takahata M. Majima T. Abe Y. Komatsu M. Iwasaki N.. (2010). Effect of selective estrogen receptor modulator/raloxifene analogue on proliferation and collagen metabolism of tendon fibroblast. Connect. Tissue Res. 51, 179–187. 10.3109/03008200903204669 20073985

[B49] Kitajima Y. Ono Y. (2016). Estrogens maintain skeletal muscle and satellite cell functions. J. Endocrinol. 229, 267–75. 10.1530/JOE-15-0476 27048232

[B50] Kjaer M. (2004). Role of extracellular matrix in adaptation of tendon and skeletal muscle to mechanical loading. Physiol. Rev. 84, 649–698. 10.1152/physrev.00031.2003 15044685

[B51] Kjaer M. Langberg H. Heinemeier K. Bayer M. L. Hansen M. Holm L.. (2009). From mechanical loading to collagen synthesis, structural changes and function in human tendon. Scand. J. Med. Sci. Sports 19, 500–510. 10.1111/j.1600-0838.2009.00986.x 19706001

[B52] Kumar S. Lata K. Mukhopadhyay S. Mukherjee T. K. (2010). Role of estrogen receptors in pro-oxidative and anti-oxidative actions of estrogens: a perspective. Biochim. Biophys. Acta Gen. Subjects 1800, 1127–1135. 10.1016/j.bbagen.2010.04.011 20434525

[B53] LaStayo P. C. Woolf J. M. Lewek M. D. Snyder-Mackler L. Reich T. Lindstedt S. L. (2003). Eccentric muscle contractions: their contribution to injury, prevention, rehabilitation, and sport. J. Orthop. Sports Phys. Ther. 33, 557–571. 10.2519/jospt.2003.33.10.557 14620785

[B54] Laurent G. J. (1987). Dynamic state of collagen: pathways of collagen degradation *in vivo* and their possible role in regulation of collagen mass. Am. J. Physiol. Cell Physiol. 252, C1–C9. 10.1152/ajpcell.1987.252.1.C1 3544859

[B55] Leblanc D. R. Schneider M. Angele P. Vollmer G. Docheva D. (2017). The effect of estrogen on tendon and ligament metabolism and function. J. Steroid Biochem. Mol. Biol. 172, 106–116. 10.1016/j.jsbmb.2017.06.008 28629994

[B56] Lee C. A. Lee-Barthel A. Marquino L. Sandoval N. Marcotte G. R. Baar K. (2015). Estrogen inhibits lysyl oxidase and decreases mechanical function in engineered ligaments. J. Appl. Physiol. 118, 1250–1257. 10.1152/japplphysiol.00823.2014 25979936

[B57] Lee C. Y. Liu X. Smith C. L. Zhang X. Hsu H. C. Wang D. Y.. (2004a). The combined regulation of estrogen and cyclic tension on fibroblast biosynthesis derived from anterior cruciate ligament. Matrix Biol. 23, 323–329. 10.1016/j.matbio.2004.07.004 15464364

[B58] Lee C. Y. Smith C. L. Zhang X. Hsu H. C. Wang D. Y. Luo Z. P. (2004b). Tensile forces attenuate estrogen-stimulated collagen synthesis in the ACL. Biochem. Biophys. Res. Commun. 317, 1221–1225. 10.1016/j.bbrc.2004.03.174 15094400

[B59] Lee H. Jerrold S. P. JongEun Y. (2015). Do oral contraceptives alter knee ligament damage with heavy exercise? Tohoku J. Exp. Med. 237, 51–56. 10.1620/tjem.237.51 26346968

[B60] Lee H. Petrofsky J. S. Daher N. Berk L. Laymon M. Khowailed I. A. (2013). Anterior cruciate ligament elasticity and force for flexion during the menstrual cycle. Med. Sci. Monit. 19, 1080–1088. 10.12659/MSM.889393 24287619 PMC3862144

[B61] Lefevre N Bohu Y. Klouche S. Lecocq J. Herman S. (2013). Anterior cruciate ligament tear during the menstrual cycle in female recreational skiers. Orthop. Traumatol. Surg. Res. 99, 571–575. 10.1016/j.otsr.2013.02.005 23764504

[B62] Ling-Ling E. Xu W. H. Feng L. Liu Y. Cai D. Q. Wen N. (2016). Estrogen enhances the bone regeneration potential of periodontal ligament stem cells derived from osteoporotic rats and seeded on nano-hydroxyapatite/collagen/poly (L-lactide). Int. J. Mol. Med. 37, 1475–1486. 10.3892/ijmm.2016.2559 27082697 PMC4866970

[B63] Liu S. H. al-Shaikh R. Panossian V. Yang R. S. Nelson S. D. Soleiman N.. (1996). Primary immunolocalization of estrogen and progesterone target cells in the human anterior cruciate ligament. J. Orthop. Res. 14, 526–533. 10.1002/jor.1100140405 8764860

[B64] Liu S. H. Al-Shaikh R. A. Panossian V. Finerman G. A. Lane J. M. (1997). Estrogen affects the cellular metabolism of the anterior cruciate ligament: a potential explanation for female athletic injury. Am. J. Sports Med. 25, 704–709. 10.1177/036354659702500521 9302481

[B65] Luo T. Kim J. K. (2016). The role of estrogen and estrogen receptors on cardiomyocytes: an overview. Can. J. Cardiol. 32, 1017–1025. 10.1016/j.cjca.2015.10.021 26860777 PMC4853290

[B66] Magnusson S. P. Hansen M. Langberg H. Miller B. Haraldsson B. Westh E. K.. (2007). The adaptability of tendon to loading differs in men and women. Int. J. Exp. Pathol. 88, 237–240. 10.1111/j.1365-2613.2007.00551.x 17696904 PMC2517312

[B67] Mamalis A. Markopoulou C. Lagou A. Vrotsos I. (2011). Oestrogen regulates proliferation, osteoblastic differentiation, collagen synthesis and periostin gene expression in human periodontal ligament cells through oestrogen receptor beta. Arch. Oral Biol. 56, 446–455. 10.1016/j.archoralbio.2010.11.001 21130420

[B68] Marturano J. E. Xylas J. F. Sridharan G. V. Georgakoudi I. Kuo C. K. (2014). Lysyl oxidase-mediated collagen crosslinks may be assessed as markers of functional properties of tendon tissue formation. Acta Biomater. 10, 1370–1379. 10.1016/j.actbio.2013.11.024 24316363 PMC4053294

[B69] McClung J. M. Davis J. M. Wilson M. A. Goldsmith E. C. Carson J. A. (2006). Estrogen status and skeletal muscle recovery from disuse atrophy. J. Appl. Physiol. 100, 2012–2023. 10.1152/japplphysiol.01583.2005 16497837

[B70] Miller B. F. Hansen M. Olesen J. L. Flyvbjerg A. Schwarz P. Babraj J. A.. (2006). No effect of menstrual cycle on myofibrillar and connective tissue protein synthesis in contracting skeletal muscle. Am. J. Physiol. Endocrinol. Metabol. 290, E163–E168. 10.1152/ajpendo.00300.2005 16131512

[B71] Miller B. F. Hansen M. Olesen J. L. Schwarz P. Babraj J. A. Smith K.. (2007). Tendon collagen synthesis at rest and after exercise in women. J. Appl. Physiol. 102, 541–546. 10.1152/japplphysiol.00797.2006 16990502

[B72] Miller B. F. Olesen J. L. Hansen M. Døssing S. Crameri R. M. Welling R. J.. (2005). Coordinated collagen and muscle protein synthesis in human patella tendon and quadriceps muscle after exercise. J. Physiol. 567, 1021–1033. 10.1113/jphysiol.2005.093690 16002437 PMC1474228

[B73] Minahan C. Joyce S. Bulmer A. C. Cronin N. Sabapathy S. (2015). The influence of estradiol on muscle damage and leg strength after intense eccentric exercise. Eur. J. Appl. Physiol. 115, 1493–1500. 10.1007/s00421-015-3133-9 25694209

[B74] Mishell D. R. Thorneycroft I. H. Nakamura R. M. Nagata Y. Stone S. C. (1972). Serum estradiol in women ingesting combination oral contraceptive steroids. Am. J. Obst. Gynecol. 114, 923–928. 10.1016/0002-9378(72)90098-1 4645131

[B75] Myer G. D. Ford K. R. Paterno M. V. Nick T. G. Hewett T. E. (2008). The effects of generalized joint laxity on risk of anterior cruciate ligament injury in young female athletes. Am. J. Sports Med. 36, 1073–1080. 10.1177/0363546507313572 18326833 PMC3407802

[B76] Nelson L. R. Bulun S. E. (2001). Estrogen production and action. J. Am. Acad. Dermatol. 45, S116–S124. 10.1067/mjd.2001.117432 11511861

[B77] Park S. K. Stefanyshyn D. J. Loitz-Ramage B. Hart D. A. Ronsky J. L. (2009). Changing hormone levels during the menstrual cycle affect knee laxity and stiffness in healthy female subjects. Am. J. Sports Med. 37, 588–598. 10.1177/0363546508326713 19174550

[B78] Pingel J. Langberg H. Skovgård D. Koskinen S. Flyvbjerg A. Frystyk J.. (2012). Effects of transdermal estrogen on collagen turnover at rest and in response to exercise in postmenopausal women. J. Appl. Physiol. 113, 1040–1047. 10.1152/japplphysiol.01463.2011 22773769

[B79] Pöllänen E. Fey V. Törmäkangas T. Ronkainen P. H. Taaffe D. R. Takala T.. (2010). Power training and postmenopausal hormone therapy affect transcriptional control of specific co-regulated gene clusters in skeletal muscle. Age 32, 347–363. 10.1007/s11357-010-9140-1 20640546 PMC2926854

[B80] Rahr-Wagner L. Thillemann T. M. Mehnert F. Pedersen A. B. Lind M. (2014). Is the use of oral contraceptives associated with operatively treated anterior cruciate ligament injury? A case-control study from the danish knee ligament reconstruction registry. Am. J. Sports Med. 42, 2897–2905. 10.1177/0363546514557240 25428957

[B81] Ramesh R. Von Arx O. Azzopardi T. Schranz P. J. (2005). The risk of anterior cruciate ligament rupture with generalised joint laxity. Bone Joint J. 87, 800–803. 10.1302/0301-620X.87B6.15833 15911662

[B82] Ramos J. E. Al-Nakkash L. Peterson A. Gump B. S. Janjulia T. Moore M. S.. (2012). The soy isoflavonegenistein inhibits the reduction in Achilles tendon collagen content induced by ovariectomy in rats. Scand. J. Med. Sci. Sports 22, e108–e114. 10.1111/j.1600-0838.2012.01516.x 22852581 PMC3482002

[B83] Reddy G. K. Lisa S.-B. Chukuka S. (2002). Enwemeka. Glycation-induced matrix stability in the rabbit achilles tendon. Arch. Biochem. Biophys. 399, 174–180. 10.1006/abbi.2001.2747 11888203

[B84] Rice C. L. Cunningham D. A. Paterson D. H. Rechnitzer P. A. (1989). Strength in an elderly population. Arch. Phys. Med. Rehabil. 70, 391–397. 2719543

[B85] Ronkainen P. H. Kovanen V. Alén M. Pöllänen E. Palonen E. M. Ankarberg-Lindgren C.. (2009). Postmenopausal hormone replacement therapy modifies skeletal muscle composition and function: a study with monozygotic twin pairs. J. Appl. Physiol. 107, 25–33. 10.1152/japplphysiol.91518.2008 19246654

[B86] Ruedl G. Ploner P. Linortner I. Schranz A. Fink C. Sommersacher R.. (2009). Are oral contraceptive use and menstrual cycle phase related to anterior cruciate ligament injury risk in female recreational skiers? Knee Surg. Sports Traumatol. Arthroscopy 17, 1065–1069. 10.1007/s00167-009-0786-0 19333573

[B87] Savage K. J. Priscilla M. C. (2002). Oral contraceptive use and exercise-induced muscle damage and recovery. Contraception 66, 67–71. 10.1016/S0010-7824(02)00320-7 12169383

[B88] Seneviratne A. Attia E. Williams R. J. Rodeo S. A. Hannafin J. A. (2004). The effect of estrogen on ovine anterior cruciate ligament fibroblasts: cell proliferation and collagen synthesis. Am. J. Sports Med. 32, 1613–1618. 10.1177/0363546503262179 15494324

[B89] Sewright K. A. Hubal M. J. Kearns A. Holbrook M. T. Clarkson P. M. (2008). Sex differences in response to maximal eccentric exercise. Med. Sci. Sports Exerc. 40, 242–251. 10.1249/mss.0b013e31815aedda 18202579

[B90] Shultz S. J. Levine B. J. Nguyen A. D. Kim H. Montgomery M. M. Perrin D. H. (2010). A comparison of cyclic variations in anterior knee laxity, genu recurvatum, and general joint laxity across the menstrual cycle. J. Orthop. Res. 28, 1411–1417. 10.1002/jor.21145 20872575 PMC2947333

[B91] Shultz S. J. Randy J. S. Beynnon B. D. (2011). Variations in varus/valgus and internal/external rotational knee laxity and stiffness across the menstrual cycle. J. Orthop. Res. 29, 318–325. 10.1002/jor.21243 20882589 PMC3176732

[B92] Shultz S. J. Sander T. C. Kirk S. E. Perrin D. H. (2005). Sex differences in knee joint laxity change across the female menstrual cycle. J. Sports Med. Phys. Fit. 45, 594–603. 10.1249/00005768-200405001-00719 16446695 PMC1890029

[B93] Shultz S. J. Schmitz R. J. Kong Y. Dudley W. N. Beynnon B. D. Nguyen A. D.. (2012a). Cyclic variations in multiplanar knee laxity influence landing biomechanics. Med. Sci. Sports Exer. 44, 900–909. 10.1249/MSS.0b013e31823bfb25 22033513 PMC12335006

[B94] Shultz S. J. Wideman L. Montgomery M. M. Beasley K. N. Nindl B. C. (2012b). Changes in serum collagen markers, IGF-I, and knee joint laxity across the menstrual cycle. J. Orthop. Res. 30, 1405–1412. 10.1002/jor.22093 22389002 PMC3371148

[B95] Siegel R. C. (1976). Collagen cross-linking. Synthesis of collagen cross-links *in vitro* with highly purified lysyl oxidase. J. Biol. Chem. 251, 5786–5792. 9402

[B96] Siegel R. C. Fu J. C. (1976). Collagen cross-linking. Purification and substrate specificity of lysyl oxidase. J. Biol. Chem. 251, 5779–5785. 9401

[B97] Sipilä S. Taaffe D. R. Cheng S. Puolakka J. Toivanen J. Suominen H. (2001). Effects of hormone replacement therapy and high-impact physical exercise on skeletal muscle in post-menopausal women: a randomized placebo-controlled study. Clin. Sci. 101, 147–157. 10.1042/cs1010147 11473488

[B98] Smith G. I. Yoshino J. Reeds D. N. Bradley D. Burrows R. E. Heisey H. D.. (2014). Testosterone and progesterone, but not estradiol, stimulate muscle protein synthesis in postmenopausal women. J. Clin. Endocrinol. Metab. 99, 256–265. 10.1210/jc.2013-2835 24203065 PMC3879672

[B99] Spangenburg E. E. Geiger P. C. Leinwand L. A. Lowe D. A. (2012). Regulation of physiological and metabolic function of muscle by female sex steroids. Med. Sci. Sports Exerc. 44, 1653–1662. 10.1249/MSS.0b013e31825871fa 22525764 PMC3422439

[B100] Svensson R. B. Mulder H. Kovanen V. Magnusson S. P. (2013). Fracture mechanics of collagen fibrils: influence of natural cross-links. Biophys. J. 104, 2476–2484. 10.1016/j.bpj.2013.04.033 23746520 PMC3672864

[B101] Taaffe D. R. Newman A. B. Haggerty C. L. Colbert L. H. de Rekeneire N. Visser M.. (2005). Estrogen replacement, muscle composition, and physical function: the health ABC study. Med. Sci. Sports Exerc. 37, 1741–1747. 10.1249/01.mss.0000181678.28092.31 16260975

[B102] Teixeira P. J. Going S. B. Houtkooper L. B. Metcalfe L. L. Blew R. M. Flint-Wagner H. G.. (2003). Resistance training in postmenopausal women with and without hormone therapy. Med. Sci. Sports Exerc. 35, 555–562. 10.1249/01.MSS.0000058437.17262.11 12673136

[B103] Torres M. J. Kew K. A. Ryan T. E. Pennington E. R. Lin C. T. Buddo K. A.. (2018). 17β-estradiol directly lowers mitochondrial membrane microviscosity and improves bioenergetic function in skeletal muscle. Cell Metab. 27, 167–179. 10.1016/j.cmet.2017.10.003 29103922 PMC5762397

[B104] Valencia A. P. Schappal A. E. Morris E. M. Thyfault J. P. Lowe D. A. Spangenburg E. E. (2016). The presence of the ovary prevents hepatic mitochondrial oxidative stress in young and aged female mice through glutathione peroxidase 1. Exp. Gerontol. 73, 14–22. 10.1016/j.exger.2015.11.011 26608809 PMC4824289

[B105] West D. W. Lee-Barthel A. McIntyre T. Shamim B. Lee C. A. Baar K. (2015). The exercise-induced biochemical milieu enhances collagen content and tensile strength of engineered ligaments. J. Physiol. 593, 4665–4675. 10.1113/JP270737 26282066 PMC4606526

[B106] Westh E. Kongsgaard M. Bojsen-Moller J. Aagaard P. Hansen M. Kjaer M.. (2008). Effect of habitual exercise on the structural and mechanical properties of human tendon, *in vivo*, in men and women. Scand. J. Med. Sci. Sports. 18, 23–30. 10.1111/j.1600-0838.2007.00638.x 17490462

[B107] Wojtys E. M. Huston L. J. Boynton M. D. Spindler K. P. Lindenfeld T. N. (2002). The effect of the menstrual cycle on anterior cruciate ligament injuries in women as determined by hormone levels. Am. J. Sports Med. 30, 182–188. 10.1177/03635465020300020601 11912085

[B108] Wojtys E. M. Huston L. J. Lindenfeld T. N. Hewett T. E. Greenfield M. L. (1998). Association between the menstrual cycle and anterior cruciate ligament injuries in female athletes. Am. J. Sports Med. 26, 614–619. 10.1177/03635465980260050301 9784805

[B109] Yao J. Brinton R. D. (2012). Estrogen regulation of mitochondrial bioenergetics: implications for prevention of Alzheimer's disease. Adv. Pharmacol. 64, 327–371. 10.1016/B978-0-12-394816-8.00010-6 22840752 PMC3970844

[B110] Yu W. D. Liu S. H. Hatch J. D. Panossian V. Finerman G. A. (1999). Effect of estrogen on cellular metabolism of the human anterior cruciate ligament. Clin. Orthop. Relat. Res. 366, 229–238. 10.1097/00003086-199909000-00030 10627740

[B111] Zhao R. Xu Z. Zhao M. (2015). Effects of oestrogen treatment on skeletal response to exercise in the hips and spine in postmenopausal women: a meta-analysis. Sports Med. 45, 1163–1173. 10.1007/s40279-015-0338-3 26003475

